# Editorial: Inherited and acquired ribosomopathies: missing puzzle pieces

**DOI:** 10.3389/fgene.2023.1194788

**Published:** 2023-04-12

**Authors:** Roberto Valli, Marianna Penzo

**Affiliations:** ^1^ Department of Medicine and Surgery and Genomic Medicine Center, University of Insubria, Varese, Italy; ^2^ Department of Medical and Surgical Sciences and Center for Applied Biomedical Research (CRBA), Alma Mater Studiorum University of Bologna, Bologna, Italy

**Keywords:** ribosome biogenesis, rare disease, ribosomopathy, RPL10, shwachman diamond syndrome, diamond blackfan anaemia, X-linked dyskeratosis congenita, T-ALL leukemia

Ribosomopathies are human diseases arising from altered ribosome biogenesis and function. The first of these conditions was described over two decades ago [X-linked Dyskeratosis Congenita (Heiss et al., 1998)]; but since then, the list keeps growing (Venturi and Montanaro, 2020). Ribosome biogenesis is an extremely energy demanding and complex cellular process, involving the coordinated activity of hundreds of different factors (proteins and non-coding RNAs) to produce structurally and functionally competent ribosomes (Kressler et al., 2017). Inherited or acquired genetic alterations underpinning ribosomopathies may involve ribosomal proteins (RP) coding genes [e.g., Diamond-Blackfan anemia–DBA (Costa et al., 2020)], ribosome assembly factors [e.g., Schwachman-Diamond Syndrome - SDS (Thompson et al., 2022)] or proteins involved in rRNA modifications [e.g., X-linked Dyskeratosis Congenita–X-DC (Penzo and Montanaro, 2018)] or processing [e.g., Cartilage-Hair Hypoplasia–CHH (Thiel and Rauch, 2011)]. Interestingly, all inherited ribosomopathies, which by definition arise as a consequence of germline mutations, have common features that are recapitulated as defects in highly proliferating tissues. Although paradoxically, in each disease the ribosome production defect has tissue-specific instead of ubiquitous effects. Even more intriguingly, most of ribosomopathies share an increased cancer susceptibility compared to the general population. Even though these hallmarks of ribosomopathies have been known for several years, the connection between these two apparently opposite features remains unknown.

Somatic genetic alterations broadly affecting ribosome biogenesis, are nowadays well known for having an active role in the onset and/or development of multiple cancer types, which can, therefore, be defined as acquired ribosomopathies (Sulima et al., 2017). This is very well matched with the increased cancer risk for patients affected by inherited ribosomopathies suggesting that de-regulation of ribosome biogenesis and function may actively contribute to malignant transformation (Penzo et al., 2019).

The cause-effect connections between the genetic defects undermining ribosome biogenesis and the hypo- or hyper-proliferative features of ribosomopathies are for the most part, poorly understood.

With this Research Topic, we aimed at adding some new pieces to the puzzle of understanding ribosomopathies, with a particular focus on the genetic, molecular and cellular alterations which are at the heart of these diseases. In the past decade scientists have made great advances in the characterization of the pathogenesis for some of these inherited disorders (like, for instance, in the case of SDS) (Warren, 2018), but for some others the scientific community is still struggling to find a nexus between the genetic defect, the outcome on ribosomes, and the downstream tissue-specific phenotype (like in the case of CHH) (Thiel and Rauch, 2011). It is worth mentioning that the technical advances made in the past two decades are just starting to bear fruit. We refer, for instance, to the development of approaches useful to define how the alterations impact ribosomal composition, structure, or function, which are necessary to understand the impact of the genetic alterations. In this Research Topic, Barozzi et al. propose an overview of the low- and high-throughput techniques which have recently become available to analyze pseudouridylation at specific sites in rRNAs, ranging from LC-MS to cryo-electron microscopy, passing through NGS. As these authors state, the qualitative and quantitative determination of pseudouridines at specific sites is the necessary first step in the path of defining the role of these modifications in physiology and pathology, here including X-DC.

One of the main hurdles remains the availability of appropriate experimental models to address the mechanistic effects of altered ribosome biogenesis in the developing organism, where the consequences of translation insufficiency are time- and cell-type- specific (Norris et al., 2021). In the effort to overcome this hurdle, Piantanida et al. developed a new cellular model of DBA, a rare autosomal dominant ribosomopathy characterized by failure of erythropoiesis, skeletal anomalies and incremented risk of developing malignancies [reviewed in (Ulirsch et al., 2018)]. To date, over 20 causative genes are known, mainly coding for RPs and *RPS19* is most frequently mutated (Ulirsch et al., 2018). The authors here focused on RPS26, which exhibits peculiar characteristics compared to other DBA relevant RPs. They generated a stable *RPS26* knockdown in HUDEP-1 cells, to recapitulate the erythroid phenotypic pattern of DBA patients bearing *RPS26* mutations. This model will be useful, in the near future, to deepen our knowledge of the DBA pathophysiology.

In addition to *in vitro* generated cellular models, the patients themselves are important sources of genetic and cellular material, necessary for the characterization and useful for disease modeling. In this Research Topic there are three different instances of the importance of studying patients. Cole et al. focused on DBA, studying a large family in which the phenotypic expression of DBA was highly variable, despite sharing the same *RPS19* mutation. Two members of the family showed no obvious clinical manifestation, highlighting the importance of other genetic, epigenetic or environmental factors in determining the phenotype. Furthermore, the authors underscored the importance of implementing cancer surveillance even in individuals with clinically mild phenotypes, as well as the benefit of long-term follow-up to identify late complications.

Taha et al. focused on SDS, in which the major causative gene is *SBDS* alone or in conjunction with mutations in other genes (Morini et al., 2019). It has been proposed that the interstitial deletion of chromosome 20, del (20) (q), or somatic mutations in *EIF6* gene in the bone marrow could play a rescue role in SDS (Tan et al., 2021). Taha et al. studied an SDS patient by Whole Exome Sequencing (WES), finding canonical *SBDS* mutations in association with germline *EIF6* mutations, thus confirming the benign effects of *EIF6* inactivating mutations in SDS patients.

Bacci et al., finally, adopted a WES approach on a cohort of pediatric patients affected with T cell Acute Lymphoblastic leukemia (T-ALL) to study the somatic mutations occurring in RPs, ribosome biogenesis factors and translation regulatory factors. In fact, there is evidence of altered ribosomes playing a role in driving the malignant transformation, for T-ALL as well as for other cancer types (Penzo et al., 2019). These authors found a novel mutation in *RPL10,* and linked it to translational impairment, further supporting the parallelism between inherited and acquired ribosomopathies.

Albeit known for many years, ribosomopathies are still poorly understood. The diversity and also the specificity of clinical manifestations in each of these diseases suggests that impaired ribosome biogenesis determines a range of effects based on the cellular context, underscoring once more the importance of having appropriate models to study these diseases.

The puzzle, unfortunately, is still far from being complete.
